# Seawater Desalination by Interfacial Solar Vapor Generation Method Using Plasmonic Heating Nanocomposites

**DOI:** 10.3390/mi11090867

**Published:** 2020-09-18

**Authors:** Zhourui Xu, Nanxi Rao, Chak-Yin Tang, Wing-Cheung Law

**Affiliations:** Department of Industrial and Systems Engineering, The Hong Kong Polytechnic University, Hung Hom, Kowloon, Hong Kong, China; joray.xu@connect.polyu.hk (Z.X.); nanxi.rao@polyu.edu.hk (N.R.); cy.tang@polyu.edu.hk (C.-Y.T.)

**Keywords:** plasmonic heating, nanorod, solar vapor generation

## Abstract

With the ever-growing demand in fresh water supply, great efforts have been devoted to developing sustainable systems which could generate fresh water continuously. Solar vapor generation is one of the promising strategies which comprise an unlimited energy source and efficient solar-to-heat generators for overcoming fresh water scarcity. However, current solar vapor generation systems suffer either from inefficient utilization of solar energy or an expensive fabrication process. In this paper, we introduced a nano-plasmonic approach, i.e., a floatable nanocompoiste where copper sulfide nanorods (Cu_2-x_S NRs) are embedded in a polyvinyl alcohol (PVA) matrix, for solar-to-vapor generation. A high solar vapor generation efficiency of ~87% and water evaporation rate of 1.270 kg m^−2^ h^−1^ were achieved under simulated solar irradiation of 1 sun. With the illumination of natural daylight, seawater was purified using Cu_2-x_S NRs-PVA gel, with high purity, as distilled drinking water. The plasmonic nanocomposites demonstrated here are easy to fabricate and highly efficient for solar vapor generation, illustrating a potential solution for future seawater desalination.

## 1. Introduction

Fresh water shortage has been considered as one of the most serious global issues in recent decades. Various countries are suffering from a water crisis due to the ever-growing needs of water supplies in agriculture, industrial activities, and population growth [1]. Paradoxically, over 71% of Earth’s surface is covered by water with an estimated amount of 1.4 × 10^9^ km^3^ [2]. Hence, refining drinkable water from oceans is one of the promising and sustainable approaches to overcome the fresh water shortage problem. However, the technology of current seawater desalination is not cost-effective and large infrastructural investment may be involved.

Seawater reverse osmosis (SWRO) [3], as the current dominant desalination method, has been utilized in numerous countries [4]. Nowadays, the unit water cost is stabilized at 0.5 USD m^−3^ with the large-scale energy consumption ranging within 3–4 kW h m^−3^. Moreover, due to the technical limitation in osmotic pressures, SWRO could only purify nearly 50% of water from seawater [2]. In recent years, solar water desalination, which relies on an economically sustainable energy source, solar irradiation, has attracted huge attention [5]. This is because purified water can be continuously generated by shining sunlight on the solar–thermal generators in bulk water, nearly without additional energy input and consumption [6].

The configuration of solar vapor generation has undergone a substantial evolution from the volumetric scheme [7], in which the solar-absorbing materials are suspended in bulk water for thorough thermalizing, to the interfacial scheme, i.e., solar-absorbing materials float on the water surface for localized interfacial heating [8]. In contrast to the volumetric scheme, the interfacial scheme can efficiently confine heat energy at the air–water interface and reduce the heat dissipation down to the bulk water, resulting in higher photothermal efficiency and an enhanced vapor production rate. Owing to the superior heat management of the interfacial-based solar evaporator, in recent years, great efforts [9] have been made by scientists to develop an interfacial solar evaporator with improved performance in water evaporation.

The overall performance of solar vapor generation is governed by interfacial heat generation and efficient water transportation. Photothermal materials, as the dominant constituent for heat generation, have induced extensive interests in the research community. A variety of photothermal materials, such as carbon-based materials [10] (e.g., graphene oxide [11,12], carbon nanotube [13], and carbonized plants tissues [14]), plasmonic nanomaterials (e.g., copper-chalcogenide [15], TiN [16], and gold nanoshells [17]), and other solar-harvesting materials (e.g., CuO nanowires [18], MoS_2_ [19], and Ti_2_O_3_ [20]), have been fabricated with the aim of enhancing the solar–thermal conversion efficiency. A series of floatable scaffolds, such as PVDF [15], cotton fabric [21], and polyvinyl alcohol (PVA) [22], which support photothermal materials and afford water transportation, has been designed and fabricated to exploit the full potential of the interfacial-based solar evaporator. However, the limited photothermal conversion rate and complex fabrication process of photothermal materials and the undesired water transportation condition have remained as the major obstacles for further development.

In this work, we report a Cu_2-x_S NRs-PVA gel approach, comprising photothermal generators (Cu_2-x_S NRs) [23] and a floatable PVA supporting base [24]. Copper chalcogenide semiconductor nanocrystals were considered as the most promising green and sustainable photothermal agents, due to their low cost, high photo-stability, and high absorption cross-section, which could efficiently convert solar energy into heat [25,26,27,28]. The hierarchical structure in PVA hydrogel, which consists of big channels, capillary channels, and micro holes, can efficiently replenish and transport water into confined areas for localized heating [22]. In addition, due to the low heat conduction rate, light density, and good hydrophilicity of PVA, the hydrogel possesses low unnecessary heat loss. A high vapor evaporation rate (~87%) was achieved under simulated 1 sun irradiation, and a high purity of evaporated water, which was verified by measuring the electrical resistance, was obtained by using natural sunlight. These results indicated a great potential of Cu_2-x_S NRs-PVA gel for future practical seawater desalination.

## 2. Materials and Methods

### 2.1. Materials

Polyvinyl alcohol (PVA, molecular weight of 54,000) was purchased from ACROS ORGANICS. Hydrogen chloride, glutaraldehyde solution (50% in H_2_O), copper chloride (CuCl_2_, 99%), and sodium sulfide (Na_2_S, ≥60%) were purchased from Sigma-Aldrich Co., Ltd., St. Louis, MO, USA. Polyethyleneimine (PEI, 30% in water) was purchased from TCI Co., Ltd., Tokyo, Japan. All chemicals were used as received. The seawater was collected at Ma Wan Island of Hong Kong.

### 2.2. Synthesis of Cu_2-x_S Nanorods (NRs)

The fabrication procedure of Cu_2-x_S NRs was followed from our previous work [23]. Briefly, 13.4 mg of CuCl_2_ was added into 100.0 mL deionized water and mixed with 2.0 mL of PEI solution (50 mg/mL) under constant stirring. Then, 1.0 mL of freshly prepared Na_2_S (0.1 M) was added into the previous solution. We then placed the reaction mixture into an 85 °C water bath for 15 min and the color of the reaction solution turned into dark green gradually, which indicated the formation of Cu_2-x_S NRs. Then, the as-prepared Cu_2-x_S NRs dispersion was transferred into an ice bath immediately and we waited for another 30 min to quench the reaction. To remove free reactants and concentrate the dispersion, centrifugation (3k15, SIGMA-ELEKTRO GmbH, Neustadt, Germany) with 8k rpm for 30 min with 75% acetone was performed. Different concentrations of the Cu_2-x_S NRs dispersion were prepared for further gel preparation.

### 2.3. Preparation of Polyvinyl Alcohol (PVA) gel/Cu_2-x_S NRs-PVA gel

Specifically, 2.0 g of PVA power was dissolved in 20.0 mL of DI water with constant stirring at 90 °C. After the PVA solution cooled down to room temperature, 250 uL of glutaraldehyde was further added. Then, 1.2 mL of HCl (1 M) and 480 uL of water or Cu_2-x_S NRs dispersion were added into the previous mixture to form blank PVA gel or Cu_2-x_S NRs-PVA gel. The gelation process continued for 2 h. To remove unwanted impurities, the obtained gel was purified against DI water for one day. The purified PVA gel/Cu_2-x_S NRs-PVA gel underwent freeze–thaw cycles for 10 times. Finally, the obtained PVA gel/Cu_2-x_S NRs-PVA gel was lyophilized and stored in a fridge before use.

### 2.4. Characterizations

The morphology and size of Cu_2-x_S NRs were measured by a transmission electron microscope (TEM, JEOL JEM-2011, Tokyo, Japan). The structure of PVA gel/Cu_2-x_S NRs-PVA gel was analyzed by a scanning electron microscope (SEM, JEOL 6490, Tokyo, Japan) and Leica microscope (DM500, Leica Microsystems GmbH, Nussloch, Germany). The UV–Vis–NIR absorbance of sample gels was measured by spectrometers (HR2000, and NIRQuest512, Ocean Optics, Inc., Delray Beach, FL, USA). The IR digital photos were captured by an FLIR camera (FLIR ONE PRO, Flir Systems, Inc., Goleta, CA, USA).

### 2.5. Solar Vapor Generation

The solar vapor generation test was performed on a homemade measuring system, comprising a simulated solar illuminator (91160 solar simulator, Newport Group, Inc., Irvine, CA, USA) and a 4 decimal electronic precision balance. Then, 40 × 25 mm weighing bottles containing either 20 mL of DI water or seawater were prepared for water evaporation and placed on the balance. The fabricated PVA gel or Cu_2-x_S NRs-PVA gel were cut to 30 mm diameter and floated in the weighing bottle. When applying the simulated solar illumination, with the intensity of 1 kW m^−2^, upon sample gels, the weight loss from the evaporation of water was recorded. For the cycling vapor generation tests, the sample gels were cooled down to room temperature and dried by absorbent paper for the next cycle to simulate the practical situation.

### 2.6. Water Purity Test

To simply examine the purity of the distilled water that originated from the solar vapor generation method, an electrical resistance test was performed. The electrical resistance was measured by using an IDM 503 multimeter (ISO-Tech, Kanagawa, Japan). To test the resistance, a certain volume of liquid was placed upon a glass slide, and by further dipping the two electrodes into the liquid with a fixed distance, the exact electrical resistance could be read by the multimeter. The electrical resistances of commercial distilled water and tap water were also tested as the controls.

## 3. Results and Discussion

**Figure 1:**
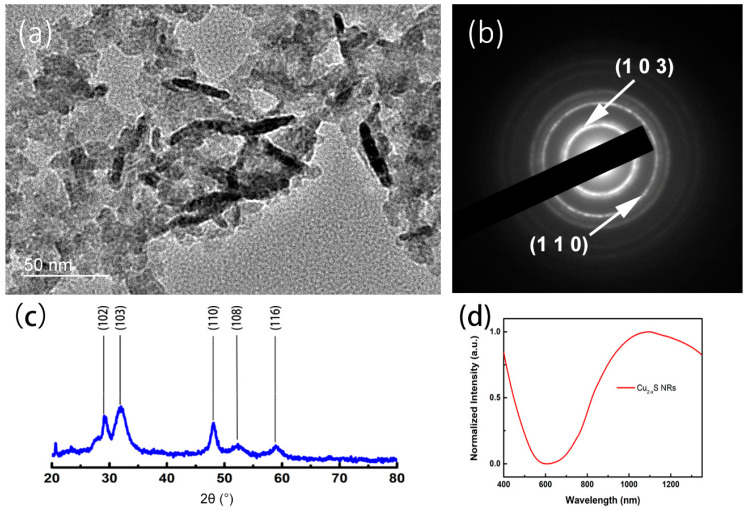
The characterization of Cu_2-x_S nanorods (NRs). The morphology and size were visualized by (**a**) TEM image. The crystal information of Cu_2-x_S NRs in selected area was illustrated by (**b**) electron diffraction pattern. (**c**) The crystal information of Cu_2-x_S NRs measured by XRD analysis. (**d**) The absorption spectra of Cu_2-x_S NRs in aqueous phase.

The cation-deficient copper chalcogenide (Cu_2-x_S) nanocrystal is a p-type semiconductor. The bandgap of most copper chalcogenide materials, including Cu_9_S_5_ [29], Cu_2-x_S nanoparticles [30], and Cu_2-x_S nanoflower [31], is ~2.0 eV and their localized surface plasmon resonance peaks are usually at the near infrared (NIR) region [32]. With the unique rod-shape structure, Cu_2-x_S NRs exhibited boarder absorption in this NIR range, resulting in stronger light absorption [33]. Briefly, under the excitation of NIR light, a collective oscillation of valence holes occurs in Cu_2-x_S nanocrystals and finally relaxes through heat generation by phonon–phonon scattering. Herein, we synthesized Cu_2-x_S NRs by a simple wet chemistry method by applying PEI as the capping and reducing agents. According to the TEM image in Figure 1a, Cu_2-x_S NRs with monodisperse shape and size were synthesized and protected by the PEI polymer. The average width and length of Cu_2-x_S NRs were 6.5 ± 2 and 38.5 ± 3 nm. It is believed that PEI polymer is responsible for the formation of rod-shaped Cu_2-x_S nanocrystals. In our previous work [23], a clear shape transition from dot to rod occurred with increasing concentration of PEI. The possible reason may be attributed to the strong affinity of the amino group of PEI to the particular crystal face of Cu_2-x_S nanocrystals at high concentration. The crystallographic phase of Cu_2-x_S NRs was further verified by the diffraction pattern in Figure 1b. The signals of the (1 0 3) and (1 1 0) planes appeared in the diffraction pattern as concentric circles, which indicates the existence of a covellite crystal phase and polycrystalline nature of Cu_2-x_S nanocrystals. The X-ray powder diffraction (XRD) patterns in Figure 1c also indicate the crystal information of Cu_2-x_S nanocrystals. Five major peaks at 29.27°, 31.78°, 47.94°, 52.71°, and 59.35° represented the (1 0 2), (1 0 3), (1 1 0), (1 0 8), and (1 1 6) phases of covellite crystal structures. In addition, the absorption spectra of Cu_2-x_S nanocrystals were measure by a UV–Vis–NIR spectrometer and are shown in Figure 1d. An intensified and broad absorption peak appeared in the NIR range. The strong light absorption property of Cu_2-x_S NRs exhibits great potential to be deployed in light-harvesting applications.

**Figure 2:**
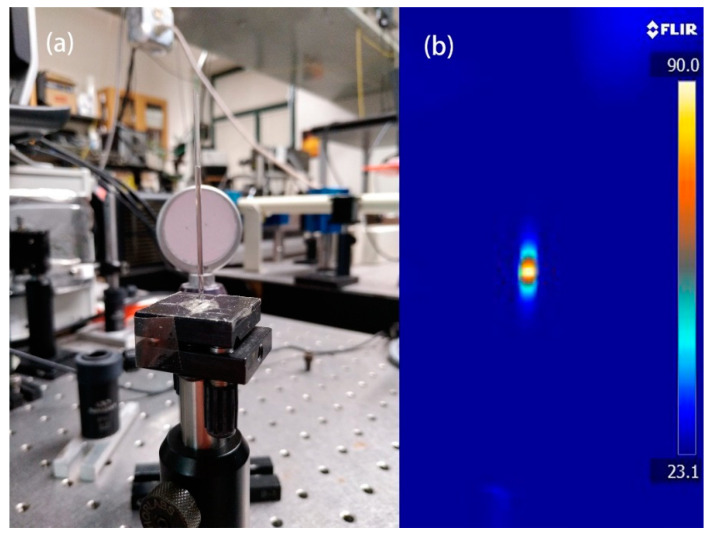
(**a**) The experiment setup to measure the temperature elevation of Cu_2-x_S NRs dispersion in a capillary tube. (**b**) The thermal image of tested Cu_2-x_S NRs sample in capillary tube at steady temperature.

Cu_2-x_S NRs, as a new type of plasmonic nanomaterial, can generate heat efficiently under light excitation. To demonstrate this property, Cu_2-x_S NRs with the concentration of 200 μg/mL were added into a capillary tube and further placed on an optic holder. The experiment setup is shown in Figure 2a. Under the illumination of NIR light for 3 min, a steady and clear temperature gradient was observed by thermal imaging. As shown in Figure 2b, a maximum temperature of 90 °C is recorded at the middle of the shining point. Generally, Cu_2-x_S NRs is a highly efficient photothermal material, which possesses great potential to be used in solar vapor generation.

The facile preparation and strong light absorption features of Cu_2-x_S NRs motivated us to study their possibility in solar vapor generation. In order to localize heat generation at the air–water interface for strengthening water evaporation, a floating base with hydrophilic nature is required. PVA, as a well-studied hydrophilic polymer with low cost, is a good candidate to fabricate a floating base. An in situ polymerization method was applied for the preparation of interconnecting structured gels. As PVA contains numerous hydroxyl groups which endow excellent hydrophilicity and negative charges and PEI-capped Cu_2-x_S NRs possess strong positive surface charges, PVA-Cu_2-x_S NRs clusters could be formed by electrostatic interaction [34]. After the gelation process, all clusters were interconnected to form the skeleton of Cu_2-x_S NRs-PVA gel. The pores between clusters formed the internal channels for water transportation.

**Figure 3:**
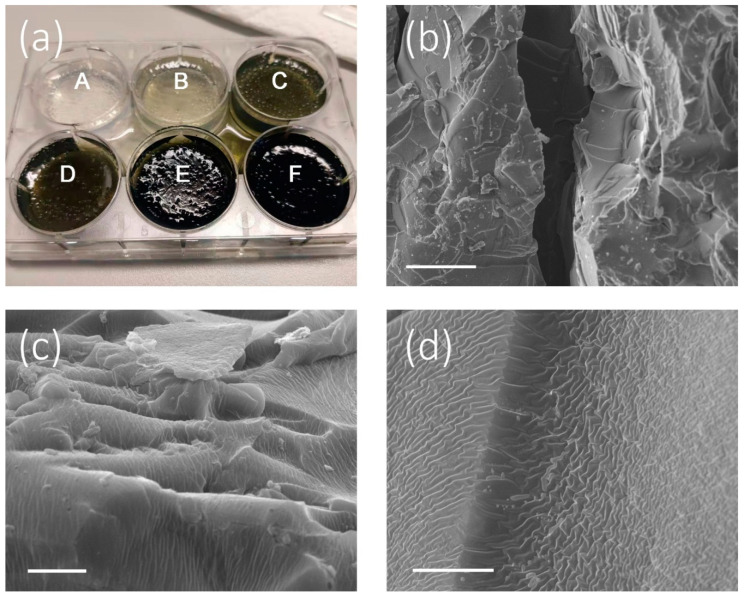
(**a**) Cu_2-x_S NRs-PVA gel with varied Cu_2-x_S NRs contents (i.e., 0, 1, 2, 4, 8, and 12 mg, which are denoted as Sample A, Sample B, Sample C, Sample D, Sample E, and Sample F). The SEM images indicate the inner hierarchical structure of Cu_2-x_S NRs-PVA gel, containing (**b**) a big channel (scale bar: 150 μm), (**c**) micro-holes (5 μm), and (**d**) mesh-typed inner wall structures (scale bar: 8 μm).

Figure 3a shows six Cu_2-x_S NRs-PVA samples with different Cu_2-x_S NRs loadings (0, 1, 2, 4, 8, and 12 mg) which correspond to Sample A, Sample B, Sample C, Sample D, Sample E, and Sample F. As shown in Figure 3a, with increasing amounts of Cu_2-x_S NRs, the color of the composites gel gradually turns to dark green from colorless. This phenomenon can be attributed to the enhanced light-harvesting ability of Cu_2-x_S NRs with increasing contents. The micro-structure of Cu_2-x_S NRs-PVA gel was further studied by SEM. As shown in Figure 3b–d, a big channel, micro-holes, and mesh-typed wall were observed in the composites gel with different magnification. According to the scale bar of 150 µm in Figure 3b, the diameter of the big channel is about 100 µm. In addition, the diameter of the micro-holes in Figure 3c is nearly 10 µm. Recently, a few reports [35,36] were published that indicated that channels with a diameter between 10 and 100 µm were suitable for water transportation. Such findings provide a solid evidence that the internal structure of the Cu_2-x_S NRs-PVA composites gel is capable of transporting water from a bulk water source to the gel surface in an efficient way. Moreover, a winkled internal surface (mesh-typed wall) is observed in Figure 3d. This surface structure was formed during the gelation and dehydration process and provided a large surface area for localized heating. Hence, the hierarchical inner structure of Cu_2-x_S NRs-PVA gel which is composed of big channels, micro-holes, and a mesh-typed wall was able to replenish water efficiently with a capillary effect and provide an extensive surface area for water thermalization.

**Figure 4:**
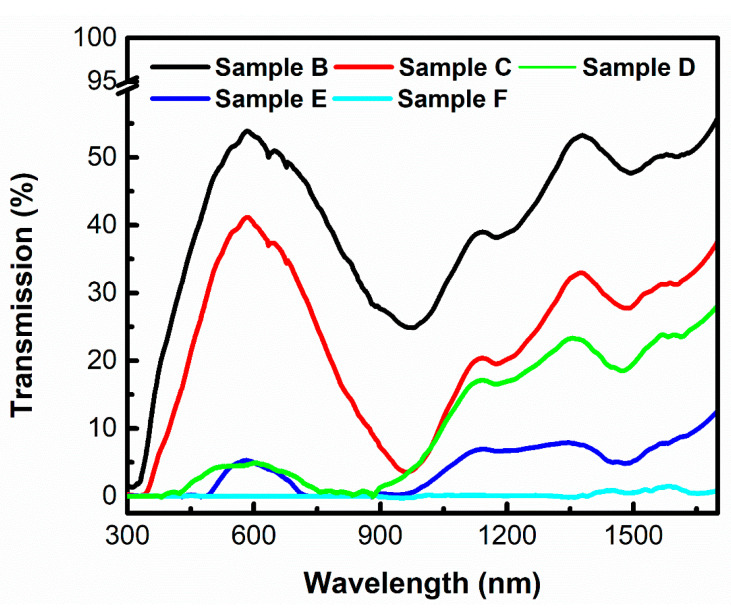
The light transmission spectra of Samples B–F with different Cu_2-x_S NRs loadings.

First, the light-harvesting material should cover a wide range of the solar energy band. Since visible and near infrared light account for over 95% of solar energy, ref. [19] the design of the solar vapor generator should cover this range as much as possible. To investigate the light absorption properties, light transmittance analysis was conducted in the wavelength range between 300 and 1700 nm. As shown in Figure 4, even with 1 mg Cu_2-x_S NRs content, nearly 50% of light was absorbed, indicating the strong light absorption property of Cu_2-x_S NRs. When the Cu_2-x_S NRs contents were increased up to 12 mg, almost no light could pass through. It is worth noting that Sample C and Sample D had similar NIR light absorption, however, there was a distinctive difference in visible light absorption. With 2 mg more of Cu_2-x_S NRs, over 95% of visible light and nearly 80% of NIR light were harvested by Sample D. However, nearly 70% of visible light and 75% of NIR light were harvested by Sample C. The absorption difference across the visible to NIR range may be attributed to the difference in the penetration depth of light with different wavelengths. It has been proved that light with a longer wavelength is able to penetrate at longer distances [37]. Hence, compared with visible light, NIR light may be more difficult to be captured by the composites gel sample. This could be the reason for the non-linear light absorption feature of the composites gel with increasing loading contents. In addition, comparing Figure 4 with Figure 1d, it can be seen that strong NIR light absorption occurred due to the existence of Cu_2-x_S NRs. However, the curves in Figure 4 contain peaks and valleys in the NIR range. These unsmoothed patterns may be attributed to the PVA polymer.

**Figure 5:**
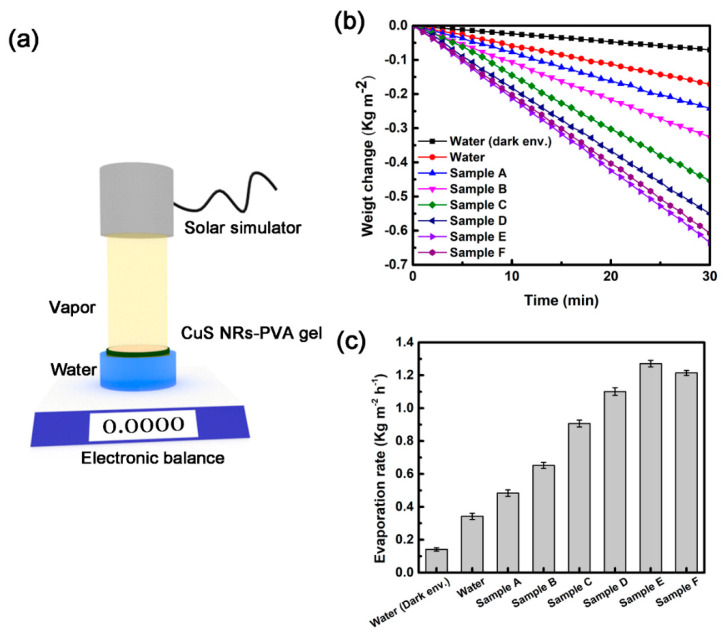
(**a**) Schematic illustration of solar evaporation. (**b**) Weight changes and (**c**) water evaporation rates of different Cu_2-x_S NRs-PVA samples under 1 sun irradiation.

To investigate the performance on solar vapor generation, Cu_2-x_S NRs-PVA gels with different Cu_2-x_S NRs loadings were tested by water evaporation experiments. The experimental setup is illustrated in Figure 5a. Briefly, a beaker of water was firstly placed on a digital balance. The Cu_2-X_s NRs-PVA gel sample was further floated on the water surface and shined by a solar simulator. The vapor production was monitored by the weight changes indicated by the digital balance. Figure 5b shows the mass loss curves of water caused by the Cu_2-x_S NRs-PVA gels under 1 sun of illumination. Numerous research works also reported that the performance of solar vapor generation increased with enhanced light intensity [6], such as 2, 4, and 10 sun of illumination. For comparison, water in a dark condition and without Cu_2-x_S NRs-PVA gel served as the control. As shown in Figure 5b, the weight changes of evaporated water increased with the amounts of Cu_2-x_S NRs and irradiation time. Specifically, Cu_2-x_S NRs-PVA gels with Cu_2-x_S NRs amounts ranging from 1 to 12 mg have a weight loss of 0.326, 0.453, 0.551, 0.635, and 0.607 kg m^−2^ of evaporated water in 30 min. The performance of 8 mg-Cu_2-x_S NRs-PVA is comparable to 12 mg-Cu_2-x_S NRs-PVA. For pure water, only a weight change of 0.171 kg m^−2^ was observed (red curve), which is far below the aforementioned results. Interestingly, the PVA gel layer could also enhance the water evaporation under 1 sun with the weight changes of 0.242 kg m^−2^ (blue curve). Such enhancement was explained by Yu et al. [22], in that the PVA matrix can change the state of the water molecule, resulting in reduced enthalpy for evaporation.

The water evaporation rates (*v*) were further calculated by using Equation (1).

(1) $$\nu =\frac{W_{loss}}{\pi {\left(\frac{D}{2}\right)}^{2}t}$$

where $W_{loss}$ corresponds to the weight losses of the evaporated water, D represents the diameter of Cu_2-x_S NRs-PVA gel, and t indicates the irradiation time period. To validate the actual contributions of Cu_2-x_S NRs-PVA gel in solar vapor generation, the water evaporation rate of pure water without sunlight irradiation was treated as the baseline and subtracted from all Cu_2-x_S NR-containing samples. The evaporation rate of Cu_2-x_S NRs-PVA gels are shown in Figure 5c. It is clear that with increasing loadings of Cu_2-x_S NRs, the performance of water evaporation gradually increased. The detailed values of the water evaporation rate are shown in Table 1. Interestingly, Sample F with higher amounts of Cu_2-x_S NRs exhibited a slightly lower water evaporation rate than Sample E. This phenomenon may be attributed to the saturation effect of photothermal materials. Hence, in view of the goal of high water conversion efficiency with economical sustainability, considering the performance in water weight loss and water evaporation rates, 8 mg-Cu_2-x_S NRs-PVA is the best candidate among the other counterparts.

**Figure 6:**
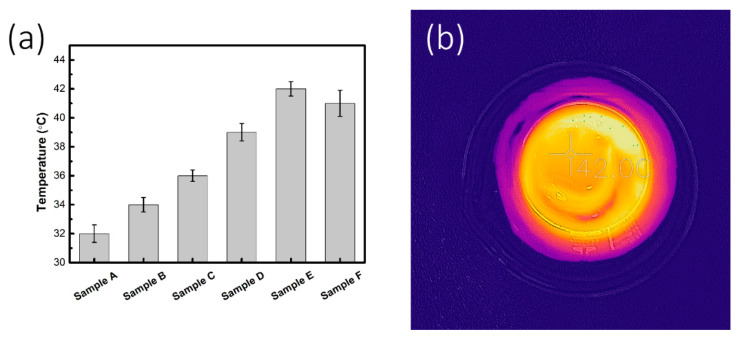
(**a**) The surface temperature of Samples A–F after 30 min of irradiation. (**b**) An IR image of Sample E under simulated 1 sun irradiation.

To study the fundamental mechanism of the enhanced water evaporation behavior, the surface temperature of Cu_2-x_S NRs-PVA composite gels was monitored under 1 sun illumination. The temperature was recorded during the irradiation tests by thermal imaging. As shown in Figure 6a, after 30 min of solar illumination of 1 sun, the surface temperatures of Samples A–F were elevated from room temperature (~23 °C) to 32, 34, 36, 39, 42, and 41 °C. In contrast to the control sample (blank PVA gel on water, 30 °C), the results indicated that Cu_2-x_S NRs can efficiently elevate the surface temperature of composite gels, hence promoting the solar-to-vapor conversion efficiency. To better illustrate the temperature gradience on the surface of Cu_2-x_S NRs-PVA composite gels, an infrared (IR) image of irradiated Sample E was captured during the solar vapor generation process, as depicted in Figure 6b.

In order to evaluate the performance of solar vapor generation, the water evaporation efficiency was calculated based on Equation (2). The water evaporation efficiency is denoted as the value of how much solar energy was utilized for vapor generation.

(2) $$\eta =\nu \frac{C\times \Delta T+\Delta_{vap}H_{m}}{C_{opt}q_{i}}$$

where *ν* represents the evaporation rate, C denotes the heat capacity of water (4.18 J g^−1^ K^−1^), ΔT is the temperature change from the initial stage (23 °C), $\Delta_{vap}H_{m}$ is the enthalpy change from liquid to vapor, $C_{opt}$ is the optical concentration, and $q_{i}$ denotes the power density of the simulated solar irradiation. For this research work, only the value of *ν* and ΔT are varied and the rest of the parameters are nearly the constant values. Hence, according to Equation (2), the water evaporation efficiencies of gel samples were calculated as Sample A (~33%), Sample B (~44%), Sample C (~62%), Sample D (~75%), Sample E (~87%), and Sample F (~83%). The detailed information is summarized in Table 1. The excellent performance of Cu_2-x_S NRs-PVA gel was attributed to the synergistic effect of the inclusion of Cu_2-x_S NRs in the PVA matrix. On one hand, the inner structures of the PVA gel enabled efficient water replenishment and localized heating; on the other hand, the great photothermal effect of Cu_2-x_S NRs can convert solar energy into thermal energy with high efficiency.

**Figure 7:**
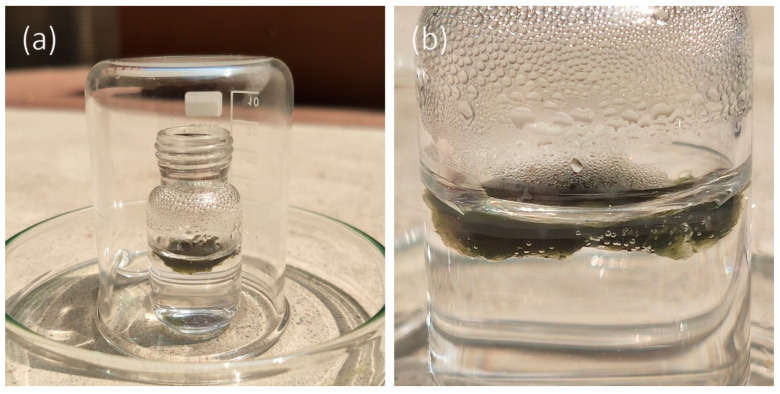
(**a**) Experimental setup of brine water desalination under natural sunlight. (**b**) The magnified view of CuS NRs-PVA gel floating on water and water droplets were condensed on the wall of the vial.

The desalination process of seawater was further performed under a practical setting. A simple homemade setup was prepared as shown in Figure 7a. A small vial containing 5 mL of seawater and a small piece of floating Cu_2-x_S NRs-PVA gel were placed on a clean petri dish, and a beaker for minimizing the influence of the surrounding environment. With illumination of natural sunlight for 4 h, water droplets appeared on the inner wall of the vial. In Figure 7b, some small air bubbles are observed at the water and Cu_2-x_S NRs-PVA gel interface, attributed to the process of water evaporation and replenishment. In fact, the performance of solar vapor generation from the field experiment was not comparable to the laboratory experiment. There are three reasons that could explain the difference in performances. First, during the outdoor experiment, the intensity of solar light could not keep constant and maximum all the time. Second, the input solar light was attenuated by the capped beaker and the glass wall of the small vial. Third, the capped beaker may afford higher humidity and the enhanced humidity may hugely inhibit the further vapor generation from the bulk water. Hence, in the future modification of the solar evaporator, the above-mentioned factors need to be taken into account.

The quality of the collected water by the solar vapor generation method is an important criterion to evaluate the reliability of the solar evaporator. To examine the quality of evaporated water, a simple but useful method was applied by measuring the electrical resistance of the water sample. Briefly, the water sample was dropped on a glass slide. Electrodes of multimeters were dipped into the liquid droplet with a fixed distance. Distilled water by the solar vapor generation method, seawater, and commercial distilled water were selected and compared to examine the purity of the water sample. An electrical resistance of 5.56 MΩ of distilled water was obtained for the evaporated water. In contrast, the electrical resistances of 0.36 and 4.46 MΩ were obtained from seawater and commercial distilled water samples under the same condition of measurement. The lower electrical resistance of seawater was attributed to the larger amounts of electrolytes. Distilled water, both from solar vapor generation and commercial water, contained less electrolytes and hence had higher purity and electrical resistance. Therefore, the aforementioned results indicated the great potential of Cu_2-x_S NRs-PVA gel for the practical desalination of seawater.

The fabrication cost of a solar evaporation device is an important issue for further commercialization [38,39]. In recent years, scientists devoted great efforts to studying and developing solar absorbers with enhanced light-harvesting ability and high solar-to-thermal conversion efficiency [40]. However, the fabrication cost, as an unavoidable issue, has always been ignored. Herein, the Cu_2-x_S NRs-PVA gels were prepared in a facile and low-cost way. With careful and detailed calculation, the production unit cost of Cu_2-x_S NRs-PVA gels is nearly USD 0.13. In addition, the prepared device can work continuously with simple maintenance procedures. However, PVA, as an environmentally friendly material, can be degraded by bacteria in a few months [41]. Although the crosslinking strategy is an efficient way to strengthen the PVA network, it is necessary to perform further modifications to improve the material endurance in the future. The novel composite gel reported in this paper is easy to fabricate, cost-effective, and highly efficient for water evaporation, illustrating a potential solution for future seawater desalination.

## 4. Conclusions

Using Cu_2-x_S NRs-PVA composite gel, high efficiency of solar vapor generation was achieved by combining the interface heating strategy with powerful photothermal agents. The water evaporation and replenishment can be enhanced by the hierarchical inner structures of Cu_2-x_S NRs-PVA gel, resulting in higher efficiency of solar to vapor conversion. Water evaporation efficiency of ~87% and solar vapor generation rate of 1.270 kg m^−2^ h^−1^ were obtained in the 8 mg-Cu_2-x_S NRs-PVA sample under 1 sun of irradiation. As demonstrated by the simple setup of the solar water purification system, the Cu_2-x_S NRs-PVA gel was capable of producing pure water under natural sunlight. The purity of evaporated water was further examined by measuring the electrical resistance. With the advantages of simple preparation steps, low fabrication cost, sustainable energy source, and high evaporation efficiency, the Cu_2-x_S NRs-PVA gel exhibits great potential for seawater desalination, and could be further extended to other applications, including environmental cooling, pollution abatement, and moisture management.

## Figures and Tables

**Table 1:** Efficiency of Cu_2-x_S nanorods-polyvinyl alcohol (NRs-PVA) gels with different Cu_2-x_S loadings under 1 sun irradiation.

Sample	Cu_2-x_S Loading Amount (mg)	υ (kg m^−2^ h^−1^)	Δ*T* (°C)	*η* (%)
A	0	0.484 ± 0.010	9 ± 0.3	33 ± 0.4
B	1	0.652 ± 0.009	11 ± 0.2	44 ± 0.5
C	2	0.906 ± 0.010	13 ± 0.2	62 ± 0.6
D	4	1.102 ± 0.011	16 ± 0.3	75 ± 0.7
E	8	1.270 ± 0.010	19 ± 0.2	87 ± 0.4
F	12	1.214 ± 0.007	18 ± 0.4	83 ± 1.1
